# An intein-mediated modulation of protein stability system and its application to study human cytomegalovirus essential gene function

**DOI:** 10.1038/srep26167

**Published:** 2016-05-18

**Authors:** Deng Pan, Baoqin Xuan, Yamei Sun, Shaowu Huang, Maorong Xie, Yadan Bai, Wenjia Xu, Zhikang Qian

**Affiliations:** 1 Unit of Herpesvirus and Molecular Virology, Key Laboratory of Molecular Virology & Immunology, Institut Pasteur of Shanghai, Chinese Academy of Sciences, University of the Chinese Academy of Sciences, Shanghai, China; 2 Institutes for Advanced Interdisciplinary Research, East China Normal University, Shanghai, China; 3 Jiangsu Key laboratory of infection and Immunity, Institutes of Biology and Medical Sciences, Soochow University, Suzhou, China

## Abstract

Functional analysis of the essential proteins encoded by human cytomegalovirus (HCMV) is hindered by the lack of complementing systems. To overcome this difficulty, we have established a novel approach, termed the intein-mediated modulation of protein stability (imPS), in which a destabilizing domain and part of a split intein are fused to the essential protein. The growth of the mutant virus can then be regulated by the degradation and splicing of the protein. We found that an ultrafast gp41-1 split intein was able to rescue or degrade the protein of interest (POI) by removing or adding a strong degron through protein splicing. As a result, the function of the POI was turned on or off during the process. Using HCMV essential gene IE1/IE2, we confirmed that imPS worked remarkably well in conditionally regulating protein stability during viral infection. This conditional approach is likely to be applicable for dissecting the gene functions of HCMV or other viruses.

Human cytomegalovirus (HCMV), the leading viral cause of birth defects, causes severe and even life-threatening diseases in immunocompromised individuals such as AIDS patients, transplant recipients, and cancer patients^1^. As a prototype β-herpesvirus, HCMV contains a 240-kb double-stranded DNA genome that potentially encodes more than 700 gene products^2^. Approximately 40 open reading frames are indicated to be essential for viral growth^3^,^4^. Dissecting the roles of these essential viral genes during viral infection is a prerequisite for the development of effective therapeutics against diseases caused by HCMV. Using bacterial artificial chromosomes (BACs) as vectors to reconstitute viruses with mutated essential genes is a powerful method for dissecting viral gene function. In most cases, the growth of the null mutant virus can be rescued later in the complementing cell lines by trans-complementation. However, it is sometimes difficult to achieve complementation simply by expressing the essential genes *in trans*, most likely because the expression patterns of the genes are important for their function and because exogenous promoters often fail to reproduce the tight regulation of endogenous promoters. Consequently, only a few essential gene mutants have been successfully complemented, and the functions of most of these essential proteins in the viral life cycle remain unknown.

Efforts have been made to address this issue. For example, a conditional approach has been developed to analyze the proteins essential to viral growth and pathogenesis^5^,^6^,^7^,^8^,^9^,^10^. This approach utilizes a mutant variant of the human FKBP12 protein (ddFKBP), which is intrinsically unstable and degrades rapidly when expressed in mammalian cells. Moreover, this protein can be stabilized by the synthetic ligand Shield-1^11^. The technique has been used to construct a recombinant virus in which a fragment encoding ddFKBP was inserted in-frame upstream to the viral essential gene of interest. The recombinant protein degraded rapidly with ddFKBP, but was restored to its normal levels in the presence of Shield-1. Hence, the recombinant virus was propagated with Shield-1. In this approach, transcriptional regulation is controlled by the endogenous promoter, and protein levels can be regulated by adjusting the Shield-1 levels. However, the 106 amino acid ddFKBP tag remains fused with the essential gene, which may disrupt the protein function. In addition, a much stronger destabilizing domain (DD) such as SopE^12^,^13^ is sometimes needed to ensure the more effective depletion of the protein of interest (POI).

In this study, we attempted to improve the system by using an ultrafast split intein. Inteins are intervening protein domains that auto-catalyze their own removal from polypeptides, and simultaneously ligate flanking extein segments together during protein splicing^14^ (Fig. 1A). In split inteins, the intein domain is divided into two pieces, an N-terminal fragment (Int^N^) and a C-terminal fragment (Int^C^), each fused with its own extein (Fig. 1A). The two halves of the split intein have an extremely high degree of affinity for each other, ensuring their instant binding and refolding into an active intein even at very low concentrations. The intact intein then mediates protein splicing *in trans*^14^,^15^,^16^.

We established a novel system, called the intein-mediated modulation of protein stability (imPS), in which protein degradation can be regulated by ultrafast protein splicing (Fig. 1B). The system’s performance in investigating essential gene functions was validated by modulating the stability and function of HCMV immediate early gene IE1/IE2 in the context of viral infection.

## Results

### Ultrafast gp41-1 split intein facilitates imPS

**Figure 1:**
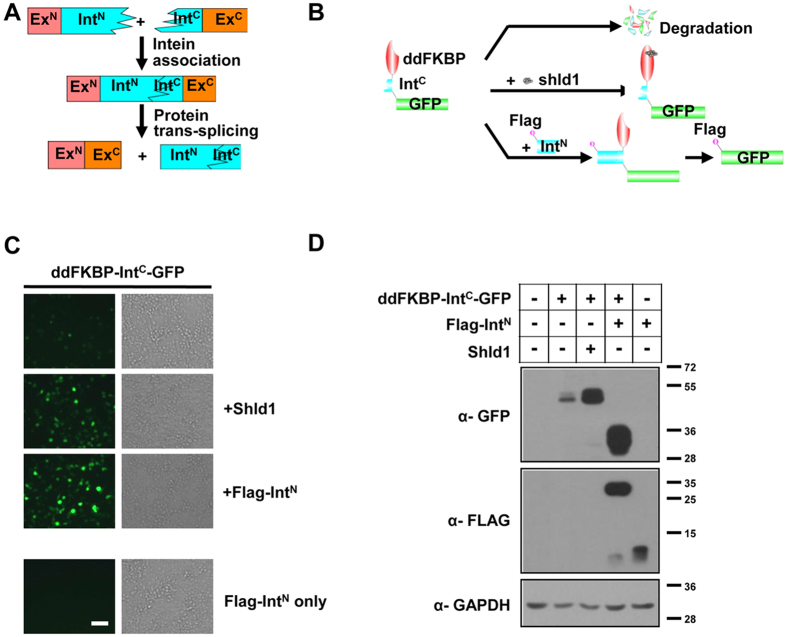
Split intein gp41-1 facilitates imPS. (**A**) Schematic diagram of protein trans-splicing. (**B**) Design and principles of imPS. A destabilization domain (such as ddFKBP) is fused to the N-terminus of Int^C^, followed by a protein of interest (such as GFP). Because the extein amino acids immediately adjacent to the intein was also important for reaching the highest rate of protein splicing, we kept the five native extein amino acids upstream and downstream of the Int^N^ and Int^C^, as described in a previously published paper^17^. (**C**) Human HEK293T cells were transfected with ddFKBP-Int^C^-GFP plasmid alone or in combination with Flag-Int^N^ or treated with 1 μM Shield-1 (Shld1). Images were taken at 48 h after transfection using fluorescence microscopy to detect the GFP signal (left panel) or cells in bright field (right panel). Scale bar = 200 μm. (**D**) HEK293T cells treated as in (**C**) and cell lysates were then analyzed by western blotting with the indicated antibodies.

It has previously been reported that the gp41-1 split intein can mediate protein splicing with an unprecedented splicing rate and efficiency *in vitro*^17^. To determine whether this intein could be used in the imPS system, a destabilizing domain derived from a mutant variant of the human FKBP12 protein (ddFKBP)^11^ was fused to the N-terminus of the C intein (Int^C^) and monitored using enhanced green fluorescent protein (GFP; Fig. 1B). The three-party fusion protein was expected to degrade in the absence of Shield-1. To rescue protein expression, we made another construct expressing Flag-tagged N intein (Flag-Int^N^). When two proteins are coexpressed, intein mediates protein splicing, excises ddFKBP-Int^C^ and Int^N^, and ligates the Flag-tag to the N-terminus of GFP, thereby rescuing GFP from ddFKBP-induced degradation (Fig. 1B). When expressing ddFKBP-Int^C^-GFP alone in HEK293T cells in this research, only a trace amount of the fusion protein was detected by western blotting (Fig. 1D, Lane 2) and fluorescence microscopy (Fig. 1C), indicating that ddFKBP works efficiently in our system. As expected, the addition of Shield-1 (1 μM) greatly increased the protein level (Fig. 1C,D, Lane 3). When coexpressed with Flag-Int^N^, the spliced Flag-GFP accumulated after protein splicing (Fig. 1C,D, Lane 4). These results indicate that ultrafast gp41-1 split intein works remarkably well in mammalian cells and can rescue the fused partner protein, suggesting that it may be appropriate for use in the imPS system to conditionally regulate protein stability and function.

### imPS works well with different types of DDs and cells

**Figure 2:**
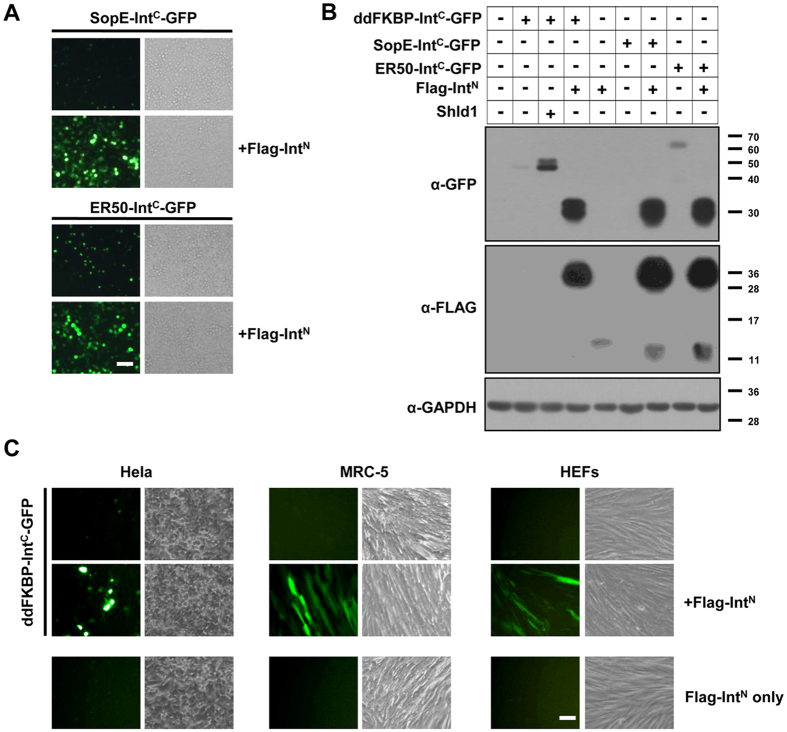
The imPS systems works well with different types of DDs and cell types. (**A**) SopE-Int^C^-GFP (*top*) or ER50-Int^C^-GFP (*bottom*) was transfected alone or together with Flag-Int^N^ into HEK293T cells and fluorescent images were taken at 48 h after transfection to show the rescue of GFP by Flag-Int^N^. Scale bar = 200 μm. (**B**) Experiments similar to those in (**A**) were performed, except with ddFKBP-Int^C^-GFP or Shld1 treatment included, as indicated. Cell lysates were analyzed by western blotting with the indicated antibodies at 48 h after transfection. (**C**) Hela cells were transiently transfected with the indicated constructs, as was done for the HEK293T cells. MRC5 and HF cells (HEFs) are primary human fibroblasts, and they were transduced with lentivirus to express ddFKBP-Int^N^-GFP alone or in combination with Flag-Int^N^. GFP expression images were taken 48 h after transfection or transduction. Scale bar = 200 μm.

A new system called split ubiquitin for the rescue of function (SURF) was recently developed to release a native protein from DDs^12^. The system is based on the chemically induced dimerization and refolding of a split ubiquitin, which subsequently recruits an ubiquitin protease to release the POI from the DD. However, the SURF system is unable to rescue protein degradation mediated by a strong DD such as SopE, possibly due to the slow kinetics of ubiquitin refolding and cleavage. We wanted to test whether the imPS system was sufficiently robust to rescue proteins from SopE-induced degradation, and we also included another DD (ER50)^18^ to determine the compatibility of imPS with other DDs. Instead of ddFKBP, SopE or ER50 was fused to the N-terminus of Int^C^ and followed by GFP. As shown in Fig. 2A,B, SopE-Int^C^-GFP was barely detectable by fluorescence microscopy or western blotting in the absence of Flag-Int^N^, and only trace amounts of ddFKBP-Int^C^-GFP and ER50-Int^C^-GFP were detected in the same conditions, which is consistent with previous reports that SopE is a strong DD that causes the complete depletion of the fusion protein 12. When the three constructs were coexpressed with Flag-Int^N^, abundant spliced Flag-GFP proteins were detected and the GFP signals were robust under fluorescence microscopy. These results show that imPS works extremely well with different DDs, and is thus likely applicable to any type of DD.

We initially used HEK293T cells for the imPS system because they are easy to transfect and work with. Subsequently, we also screened three other cells, Hela cells, human fetal lung fibroblast (MRC5) cells, and human foreskin fibroblast (HF) cells, which are routinely used in our lab. As with the HEK293T cells, almost no GFP fluorescence was detected in any of the other three cell types when ddFKBP-Int^N^-GFP was expressed alone, whereas a strong GFP signal was observed when Flag-Int^N^ was coexpressed (Fig. 2C), indicating that imPS is compatible with different cell types.

### imPS rescues protein expression when the DD is fused to the C-terminus

**Figure 3:**
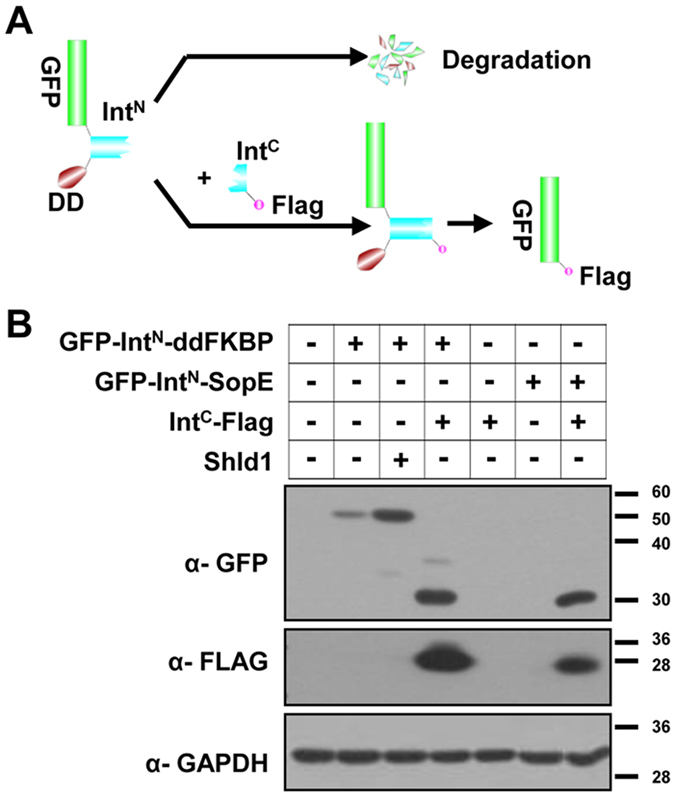
imPS rescues protein expression when a DD is fused to the C-terminus of the protein. (**A**) Experi-mental design. (**B**) Plasmid expressing GFP-Int^N^-ddFKBP or GFP-Int^N^-SopE were transfected into HEK293T cells in isolation, together with the Int^C^-Flag-expressing plasmid, or treated with Shld1 as indicated. Cell lysates were analyzed by western blotting with the indicated antibodies at 48 h after transfection to show the rescue of GFP.

We anticipated that imPS would work equally well whether the DD was fused to the N- or C-terminus of the POI and that its ability to modulate protein stability by fusing the DD to either terminus would be a desirable feature in certain circumstances. Overlapping genes may result in one terminus being unavailable for fusion. In addition, proteins undergo proteolytic processing during maturation, and a mature protein may stabilize if the DD is attached at the other end. For example, the N terminus of pUL77, an essential viral protein needed for HCMV genome encapsidation^19^, has been found essential for its function, meaning that ddFKBP can be tagged only at the C terminus^5^. However, it has also been reported that ddFKBP fused at the N-terminus of the POI works better than that fused at the C-terminus^11^. To test imPS in such circumstances, we fused Int^N^ and either ddFKBP or SopE to the C-terminus of GFP and engineered a construct for expressing Int^C^ with a Flag-tag at the C-terminus (Fig. 3A). A small amount of GFP-Int^N^-ddFKBP was detected when expressed alone, but no GFP-Int^N^-SopE was detected (Fig. 3B). These results are consistent with SopE being a stronger DD than ddFKBP^12^. The addition of Shield-1 stabilized GFP-Int^N^-ddFKBP, whereas coexpressing Int^C^-Flag induced protein splicing and rescued both ddFKBP- and SopE-tagged GFP (Fig. 3B). imPS therefore worked efficiently with DDs fused to the C-terminus of the POI.

### imPS can induce protein degradation by tagging a DD to a POI

**Figure 4:**
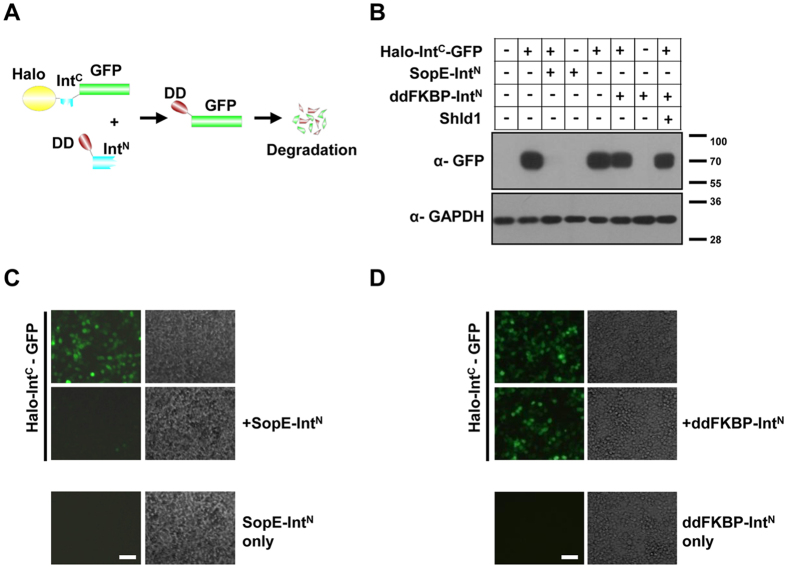
imPS induces protein degradation by tagging the DD to the protein of interest. (**A**) Experimental design. (**B**) Halo-Int^C^-GFP was transfected into HEK293T cells alone or together with SopE-Int^N^ or FKBP-Int^N^, and cell lysates were analyzed by western blotting with the indicated antibodies at 48 h after transfection to show the degradation of GFP. (**C**,**D**) Fluorescent images were taken 48 h after transfection to show the depletion of GFP by SopE-Int^N^ (**C**) or FKBP-Int^N^ (**D**) coexpression. Scale bar = 200 μm.

Having shown that imPS can successfully rescue proteins from degradation, we next tested whether it is also able to induce protein degradation by tagging a DD to the POI. We engineered another three constructs, one for the expression of GFP with an N-terminal Halo-tag (a mutated hydrolase designed to bind covalently to synthetic ligands that can be attached to various functional groups, allowing the rapid detection and efficient purification of its fusion protein^20^) and Int^C^ and two for the expression of ddFKBP or SopE fused to the N-terminus of Int^N^ (Fig. 4A). When Halo-Int^C^-GFP was expressed alone, it was detected by both fluorescence microscopy and western blotting (Fig. 4B–D). Surprisingly, when Halo-Int^C^-GFP was coexpressed with ddFKBP-Int^N^, we observed no induced degradation of Halo-Int^C^-GFP (Fig. 4B,D), possibly because of poor ddFKBP-Int^N^ expression or because ddFKBP fused at the N-terminus of Int^N^ may interfere with its splicing activity. Because the antibodies against ddFKBP or Int^N^ were unavailable, we decided not to pursue this issue further. However, when Halo-Int^C^-GFP was coexpressed with SopE-Int^N^, GFP was almost completely depleted (Fig. 4B,C). This result suggests that when SopE is used as a DD, imPS is capable of inducing protein degradation with a high degree of efficiency.

### imPS can be used to regulate the stability of essential viral gene expression

**Figure 5:**
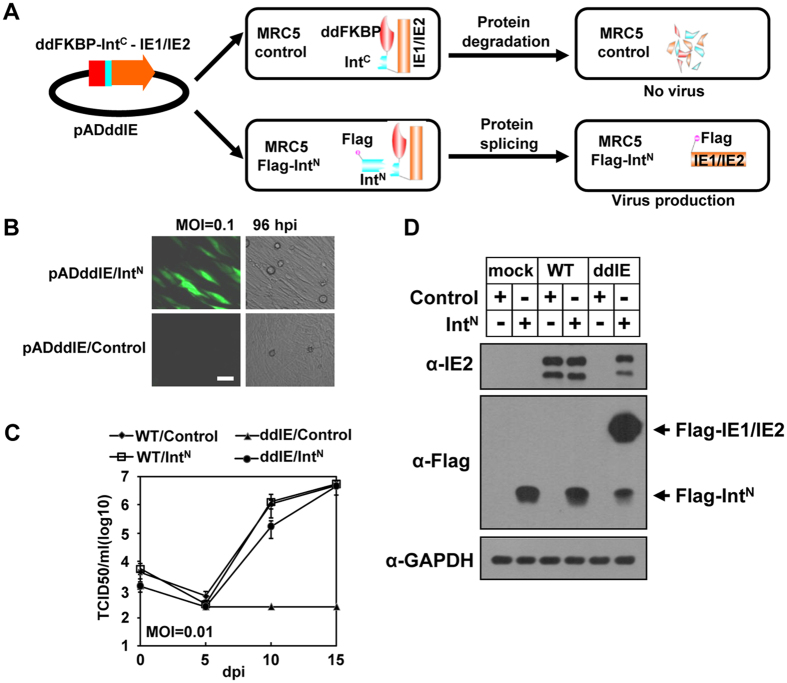
imPS can be used to regulate the stability of essential virus proteins during viral infection. (**A**) Diagram showing the use of imPS to regulate HCMV IE1/IE2 gene expression. The pADddIE construct was made by BAC recombination in which a ddFKBP degron followed by Int^C^ was fused to the N-terminus of IE1/IE2. In the MRC5 control cells, the fusion protein was degraded. Because IE1/IE2 is essential for HCMV growth, a virus could not be made in these cells. When the recombinant virus genome was transfected into the MRC5 cells expressing Flag-Int^N^, the protein splicing rescued IE1/IE2 because Flag-tagged proteins, and hence the virus, could be produced. (**B**) ADddIE virus, in which the IE1/IE2 gene was fused with ddFKBP and Int^C^ at its N-terminus, infected MRC5 control cells, or Flag-Int^N^-expressing cells at an MOI of 0.1, and the GFP signal observed using fluorescence microscopy. (**C**) A multiple-step growth curve was performed to examine the growth kinetics of the wild type (WT) virus or recombinant virus (ADddIE). ADddIE failed to grow in the control cells (ddIE/Control), whereas Flag-Int^N^ expression rescued growth to almost WT levels (ddIE/Int^N^). However, there was no difference in WT virus growth in the control (WT/Control) and Flag-Int^N^-expressing cells (WT/Int^N^). (**D**) WT or ADddIE (ddIE) virus was infected at an MOI of 1 in control or Flag- Int^N^-expressing cells (Int^N^), and cell lysates were collected at 24 h post infection and analyzed by western blotting with the indicated antibodies.

We next tested the usefulness of imPS by applying it to modulate the stability and function of HCMV immediate early gene IE1/IE2, which is known to be important for HCMV^1^,^21^. We first generated a recombinant HCMV genome (pADddIE) in which ddFKBP and Int^C^ were inserted before the IE1/IE2 open reading frame (Fig. 5A, Supplementary Fig. 1A,B). The virus was reconstituted in MRC5 cells expressing Flag-Int^N^ and analyzed using vector-only control or MRC5-Flag-Int^N^ cells. With the GFP cassette built into the virus genome, GFP expression can be used as a convenient signal for virus replication. When infected with a low multiplicity of infection (MOI), GFP expression was clearly observed in the MRC5-Flag-Int^N^ cells, whereas almost none was detected in the MRC5-control cells (Fig. 5B).

The next step was to measure the growth kinetics of the parental virus and recombinant virus ADddIE in the MRC5-control and MRC5-Flag-Int^N^ cells. As shown in Fig. 5C, the growth of the wild type (WT) virus in MRC5 expressing Flag-Int^N^ was similar to that in the MRC5-control cells, indicating that Flag-Int^N^ expression does not affect WT virus growth. In stark contrast, the recombinant virus ADddIE failed to grow in the MRC5-control cells (ddIE/Control) throughout the 15 days of the experiment, although growth was comparable with the WT virus in the MRC5-Flag-Int^N^ cells (ddIE/Int^N^). The spliced Flag-IE1/IE2 were also detected via western blot (Fig. 5D). These results showed that imPS is successful at controlling the stability, and hence function, of IE1/IE2.

### imPS can be used to study the functions of IE1/IE2 during viral infection

**Figure 6:**
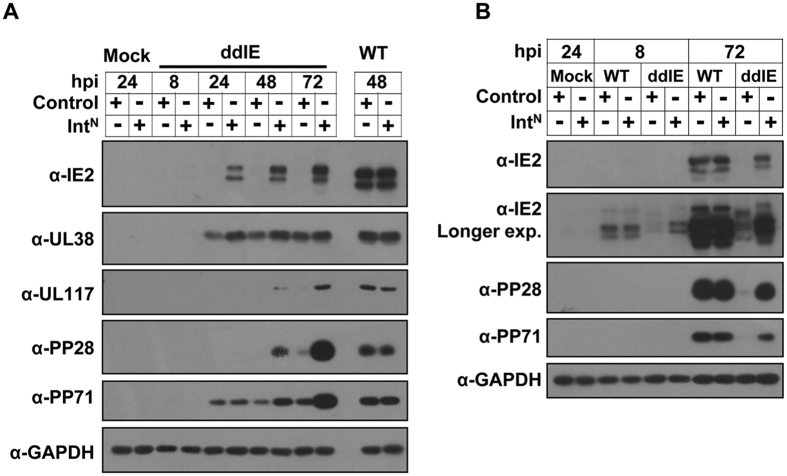
The effects of IE1/IE2 on the expression levels of HCMV proteins after virus infection. WT virus or ADddIE (ddIE) recombinant virus was infected at an MOI of 1 in control or Flag- Int^N^-expressing cells (Int^N^); cell lysates were collected after virus infection at the time points indicated and analyzed by western blotting with the indicated antibodies.

Using the imPS system, we investigated some of the IE1/IE2 functions in the context of infection. We first examined the expression levels of the HCMV viral proteins after recombinant virus ADddIE infection. As the growth curve results discussed above showed, the protein levels were similar when WT virus infected both cell types at different times (Fig. 6A,B), indicating that the expression of Flag-Int^N^ does not affect the protein expression of the WT virus. As expected, almost no IE1/IE2 expression was detected during ADddIE virus infection in the MRC5-control cells, but the protein was rescued to near WT levels in the MRC5-Flag-Int^N^ cells (Fig. 6A,B). Consistent with the critical role of IE1/IE2 in trans-activating early and late virus gene expression, the expression of early protein UL117 and late proteins pp71 and pp28 were greatly reduced in the ADddIE-infected control cells, whereas their expression was rescued almost to WT levels in the MRC5-Flag-Int^N^ cells (Fig. 6A,B). Surprisingly, although slightly reduced, a remarkable amount of early protein UL38^22^ accumulated in the absence of IE1/IE2 (Fig. 6A).

IE2 is reported to be of significant importance for the expression of some HCMV early genes^23^. Recent research showed eight proteins of the HCMV strain Merlin—UL27, UL29, UL135, UL138, US2, US11, US23, and US24—to exhibit peak expression at 6–18 hours after infection, which was earlier than previously reported^24^. We therefore examined the expression of UL27, UL29, US2, US11, US23, and US24 (the laboratory-adapted AD169 strain used in our lab lacks UL135 and UL138) using RT-qPCR to determine whether their expression was affected by the degradation of IE1/IE2. As the results shown in Fig. 6A suggest, such degradation had little effect on the protein level of UL38, although that of UL117 was remarkably reduced. Hence, the mRNA levels of UL38 and UL117 were also examined as a control.

**Figure 7:**
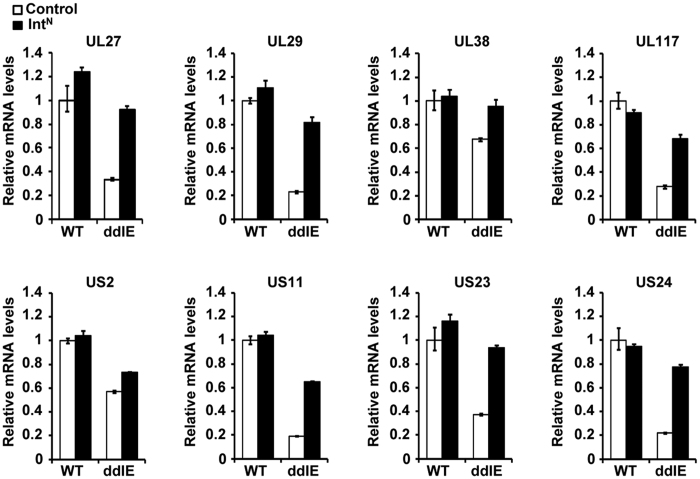
The effects of IE1/IE2 on the mRNA levels of some HCMV proteins appear early in infection. WT virus or ADddIE (ddIE) recombinant virus was infected at an MOI of 0.1 in control or Flag- Int^N^-expressing cells (Int^N^), and cell lysates were collected at 24 h post infection. Relative mRNA levels were determined by qRT-PCR with primers specific to the set of viral genes. The results were normalized to GAPDH, and the mean values with standard errors from two biological and two technical replicates are shown.

MRC5-control cells and Flag-Int^N^ cells were transfected with the WT virus or ADddIE virus with an MOI of 0.1 and cultured for 24 hours, after which total RNA was prepared from the cultures and used to perform RT-qPCR analysis. As expected, the relative mRNA level of all these genes remained similar regardless of whether the WT virus-infected MRC5-control cells or Flag-Int^N^ cells were used. However, there was some difference between the ADddIE virus-infected control cells and Flag-Int^N^ cells (Fig. 7). The transcription levels of UL117, UL27, UL29, US11, US23, and US24 decreased sharply in the ADddIE-infected control cells, and were almost equal to the WT levels in the MRC5-Flag-Int^N^ cells. However, UL38 and US2 mRNA underwent only a slight reduction with the degradation of IE1/IE2.

## Discussion

Reconstituting viruses with mutated essential genes is a powerful method of dissecting viral gene function, and a complement system is critical for the growth of mutated viruses. However, it is sometimes difficult to make a complementing cell line because (*1*) the viral gene is toxic when expressed in isolation; (*2*) primary cells permissive for viral infection may not form stable cell lines; and (*3*) the exogenous promoter may not generate the appropriate timing and expression levels.

In this study, we established a novel approach (imPS) by combining the induced-protein-degradation and ultrafast protein splicing techniques to study essential viral gene function. As shown in Fig. 1B, in the imPS system, the POI is depleted in a fast and efficient manner by fusing the DDs. An even faster protein splicing event could reverse the process before the protein degrades. Here, we show that the gp41-1 split intein (*1*) rescues proteins from ddFKBP-induced degradation almost as well as its ligand Shield-1 does; (*2*) is able to rescue proteins from more potent SopE-induced protein degradation; (*3*) works very efficiently in several different human cell types; (*4*) rescues proteins when DDs fuse to the C-terminus of the POI; and (*5*) is capable of tagging a POI with SopE via trans-splicing, which then induces its degradation. These results establish that the gp41-1 split intein is ideal for use in imPS.

The advantages of imPS over the existing approaches used in viral gene functional studies are significant. First, no small molecules, which are expensive and may otherwise interfere with the cellular pathways, are required. Second, protein splicing replaces the large DD with a smaller tag that is far less likely to adversely affect protein function. Third, most tunable DD systems are restricted to tagging at the N-terminus of the protein to ensure efficient protein degradation, but imPS allows the tagging of SopE at both termini.

As with all novel experimental techniques, imPS has room for improvement. First, although small, the Flag-tag may still affect protein function. However, as imPS can be used to tag DDs at either terminus of the POI, that event is unlikely to be a problem for many POIs. Furthermore, modified inteins can mediate N- or C-cleavage without adding any extra amino acids to the extein^17^,^25^, and those modified inteins can be incorporated into imPS. Second, the approach is not reversible, as Shield-1 is controlled. It could be turned into a more powerful system in the future if we could manage to rapidly turn on or off the activity of the intein, such as by delivering part of it (Int^N^ or Int^C^) directly into the cell with protein transduction domains (PTDs)^26^.

To evaluate the utility of imPS for studying HCMV essential gene functions, we successfully generated a mutant HCMV virus, ADddIE, containing ddFKBP and Int^C^ fused at the N-terminus of HCMV essential gene IE1/IE2. The HCMV IE2 protein is tightly regulated during infection^27^, and its expression in isolation arrests the cell cycle, making it impossible to obtain a stable cell line^28^. ADddIE was barely capable of growing in normal cells, but was able to grow to almost WT levels in the Int^N^ expressing cells (Figs 5 and 6), suggesting that imPS works well for the conditional knockdown of IE1/IE2. We also used this mutant virus (ADddIE) to investigate some of the functions of IE1/IE2. We found that during viral infection, the protein levels of both early and late genes are regulated by IE1/IE2 (Fig. 6). Only the expression of UL38 underwent few changes with IE1/IE2 degradation at both the protein and mRNA levels (Figs 6 and 7), although a previous report indicated that the UL38 gene is expressed with early kinetics as UL38 RNA is significantly reduced in the presence of anisomycin (protein synthesis inhibitor)^22^. This result suggests that the expression of UL38 is probably controlled by other immediate early proteins rather than by IE1/IE2. We also found that the transcription of UL27, UL29, US11, US23, and US24, which have been reported to appear early during HCMV infection^24^, was regulated by IE1/IE2, whereas that of US2 underwent an insignificant change with the depletion of IE1/IE2, indicating that US2 may be expressed with immediate early kinetics.

In the future, we may be able to use imPS to conduct further research on the functions of IE1/IE2 or other HCMV essential genes during infection. For example, as imPS worked efficiently with SopE fused to the C-terminus of the POI, and the growth of the WT virus was not affected by the expression of Int^C^ (Supplementary Fig. 1C), we could degrade IE2 only by tagging SopE at the C-terminal of IE2 because the last exon of IE1 and IE2 was different.

In conclusion, we have developed and demonstrated a novel system (imPS) for regulating protein stability and function *in vivo* by combining DD-induced protein degradation and ultrafast protein splicing techniques, and have successfully applied it to regulate HCMV immediate-early proteins IE1/IE2 during viral infection. The imPS system may facilitate the study of the gene functions of HCMV and other viruses. Furthermore, it has the potential to create life-attenuated viruses for vaccine development. Therefore, imPS could become a valuable tool for both basic and translational research into viral infection and related areas.

## Methods

### Plasmids and reagents

GFP fragment was amplified from pEBNA-GFP (pYD-C160)^6^. ddFKBP fragment was amplified from pENTR221-FKBP^29^. Split intein gp41-1, SopE and ER50 coding sequence (Supplementary Fig. 2) were synthesized by Nanjing Genescript Biotechnology. The following DNA fragments encoding ddFKBP-Int^C^-GFP, Flag-Int^N^, SopE-Int^C^-GFP, ER50-Int^C^-GFP, GFP-Int^N^-ddFKBP, GFP-Int^N^-SopE, Int^C^-Flag, ddFKBP-Int^N^,, SopE-Int^N^, Halo-Int^C^-GFP were amplified by overlapping PCR, and digested with SalI and EcoRI restriction enzymes, then subcloned into pLKO-dcMV-tetO carrying the CMV-TetO2 promoter (generous gifts from Roger Everett, University of Glasgow Centre for Viral Research)^30^. Primer sequences for overlapping PCR were available upon request.

Primary antibodies used in this study include: anti-GFP (7G9, Abmart), anti-Flag (3B9, Abmart), anti-GAPDH (Hangzhou Goodhere Biotechnology), anti-UL117^31^, anti-IE2 (a gift from Jay Nelson at Oregon Health and Science University), anti-pp28, anti-pp71, and anti-UL38 (gifts from Thomas Shenk at Princeton University). Shld1 was purchased from Cheminpharma (Farmington, CT)^7^.

### Cells and viruses

HEK293T cells, primary human embryonic lung fibroblasts (MRC5), primary human foreskin fibroblasts (HF) and Hela cells were propagated in Dulbecco’s modified Eagle medium (DMEM) supplemented with 10% fetal bovine serum (FBS).

Plasmid transfection into HEK293T cells or Hela cells were carried out by using polyethylenimin (PEI) as described^32^. To produce pLKO-based lentiviruses, HEK293T cells were transfected with corresponding pLKO vectors along with pVSV-G (expressing the vesicular stomatitis virus envelope protein) and pCMV-Δ9.2 (expressing all necessary lentivirus helper functions)^30^. Lentivirus stocks were collected at 48 and 72 hours after transfection, and used to transduce MRC5 or HF cells as described previously^33^.

Two BAC-HCMV clones were used in this study to reconstitute recombinant HCMV viruses. pAD-GFP carried the GFP-tagged genome of the HCMV AD169 strain and was used to produce wild-type virus^31^. pADddIE was used to reconstitute ADddIE, the mutant virus in which ddFKBP-Int^C^ was engineered before IE1/IE2 to specifically modulate IE1/IE2 expression. To create pADddIE, we generated a cassette that contained the FRT-bracketed kanamycin selection marker (amplified from pYD-C191^34^) and a downstream ddFKBP-Int^C^ fragment (amplified from pLKO-ddFKBP-Int^C^-GFP) by overlap PCR with primer sets as follows: Forward Pri: 5′-ACT GTT CCT TTC CAT GGG TCT TTT CTG CAG TCA CCG TCC TTG ACA CGA TGA AGG ACG ACG ACG ACA AGT AA-3′, internal Reverse Pri: 5′-ACC ACG TCG TGG AAT GCC TTC-3′, internal Forward Pri: 5′-GAA GGC ATT CCA CGA CGT GGT ATG GGA GTG CAG GTG GAA AC-3′, and Reverse pri: 5′-GGG CCC TCG TCA GGA TTA TCA GGG TCC ATC TTT CTC TTG GCA GAG GAC TCC ACG TCG CTG CTG CTG CTG TT-3′. The 50-bp sequences at the 5′ end of Forward and Reverse primers were homologous to the viral DNA sequences immediately upstream or downstream of the start codon of the UL123 ORF (IE1). The cassette was recombined into the IE1/IE2 gene in pAD-GFP, and the kanamycin sequence was subsequently removed by Flp/FRT recombination as previously described^7^. The final clone (pADddIE) contained ddFKBP-Int^C^ expression in frame of IE1/IE2.

To reconstitute the virus, 2 μg of the BAC-HCMV DNA and 1 μg of the pp71 expression plasmid were transfected into MRC5 or MRC5-Flag-Int^N^ cells by electroporation as described previously^35^. Culture medium was changed 24 h later, and the recombinant virus was prepared by harvesting cell-free culture supernatant when the entire monolayer of infected cells was lysed. Alternatively, virus stocks were produced by collecting cell-free culture media from infection at the multiplicity of infection of 0.05. Virus titers were determined by 50% tissue culture infectious dose (TCID50) assay in MRC5-Flag-Int^N^ cells.

### Western Blotting Analysis

Proteins were analyzed by immunoblotting. Briefly, cells were collected and washed with PBS once, then lysed in the lysis buffer (50 mM Tris [pH 7.5], 150 mM NaCl, 1% Triton X-100, protease inhibitors (Complete, EDTA-free, Roche), 10 mM NaF, 1 mM Na3VO4, 25 mM Beta-glycerophosphate, 1 mM PMSF) at 4 °C for 15 min, then centrifuged at 12,000 rpm at 4 °C for 5 min. In some cases, protein samples were collected directly in 3× SDS sample buffer. Proteins from equal cell numbers were separated by electrophoresis on a SDS-containing polyacrylamide gel, transferred to a PVDF membrane, hybridized with primary antibodies, reacted with HRP-conjugated secondary antibodies, and visualized by Clarity Western ECL Substrate (Bio-Rad).

### Microscopy analysis

The transfected cells or transduced cells were observed with a leica fluorescence microscope (Leica DMI 3000 B). Images were captured with a Leica DFC 500 digital camera.

### Analysis of viral growth kinetics

MRC5 control or MRC5-Flag-Int^N^ cells were seeded in 12-well dishes overnight to produce a subconfluent monolayer. Cells were then inoculated with recombinant HCMV viruses for 1 h at an MOI of 0.01 for multistep growth analysis. The inoculum was removed, the infected monolayers were rinsed with phosphate-buffered saline (PBS), and fresh medium was replenished. At various times post infection, cell-free virus was collected from cells by harvesting medium from infected cultures and was titered by 50% tissue culture infectious dose (TCID50) assay in MRC5-Flag-Int^N^ cells.

### RNA analysis

Relative mRNA levels were analyzed by reverse transcription coupled to real-time quantitative PCR (RT-qPCR) as previously described^36^. Total RNA was extracted using the Trizol reagent (Invitrogen). cDNA was reverse transcribed with oligo dT primer and random 6 mers using primerscript RT reagent kit (TaKaRa). cDNA was then quantified using SYBR Premix Ex Taq (TaKaRa) with primer pairs specific for viral genes UL27, UL29, UL38, UL117, US2, US11, US23, US24, or the GAPDH (glyceraldehydes-3-phosphate dehydrogenase) cellular gene. The sequences of RT-qPCR primers were listed in Supplementary Table 1. All reactions were performed in two biological and two technical replicates using an Applied Biosystems 7900HT Real-Time PCR System. Results were normalized to GAPDH.

## Additional Information

**How to cite this article**: Pan, D. *et al.* An intein-mediated modulation of protein stability system and its application to study human cytomegalovirus essential gene function. *Sci. Rep.* **6**, 26167; doi: 10.1038/srep26167 (2016).

## Supplementary Material

Supplementary Information
