# Supplementary material for: An intein-mediated modulation of protein stability system and its application to study human cytomegalovirus essential gene function
Source: Sci Rep. 2016 May 18;6:26167. doi: 10.1038/srep26167 (PMC4870628; doi:10.1038/srep26167)
Supplement: Supplementary Information [file srep26167-s1.pdf]

An intein mediated modulation of protein stability system to study human cytomegalovirus essential gene function

Deng Pan,[1,+] Baoqin Xuan,[1,+] Yamei Sun,[1] Shaowu Huang,[1,2] Maorong Xie,[1,3] Yadan Bai,[1,3] Wenjia Xu,[1] and Zhikang Qian [1,*]

[1] Unit of Herpesvirus and Molecular Virology, Key Laboratory of Molecular Virology & Immunology, Institut Pasteur of Shanghai, Chinese Academy of Sciences, Shanghai, China

[2] Institutes for Advanced Interdisciplinary Research, East China Normal University, Shanghai, China

[3] Jiangsu Key laboratory of infection and Immunity, Institutes of Biology and Medical Sciences, Soochow University, Suzhou, China

[*] Corresponding author: zkqian@ips.ac.cn (ZQ)

[+] These authors contributed equally to this work

Supporting Information

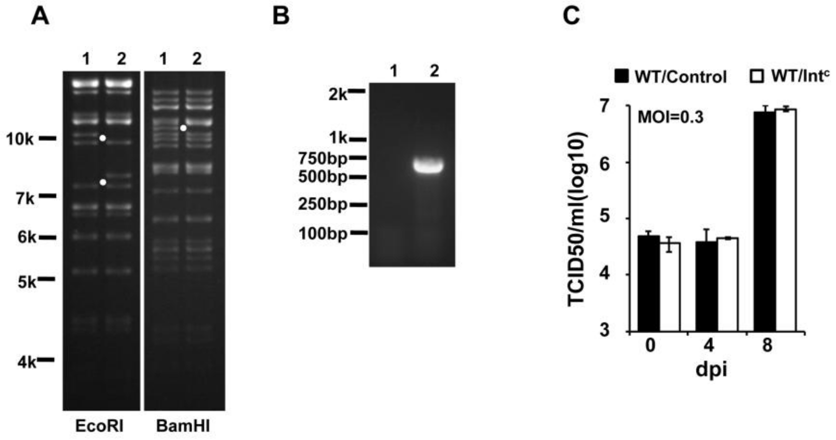

Supplementary Figure 1. Construction of pADddIE recombinant virus. (A) Restriction digestion of WT (1) and pADddIE (2) virus BAC genome by EcoRI or BamHI showed the expected bands, indicated by white dots. (B) PCR amplification using one primer corresponding to the ddFKBP-Int^{C} coding sequence and the other from the IE1/2 coding sequence gave bands for pADddIE-containing virus only. (C) A multiple step growth curve was performed to examine the growth kinetics of wild type (WT) on MRC-5 control cells (WT/Control) or Int^{C}-flag-expressing cells (WT/Int^{C}).

gp41
acccgcagcggctactgcctggacctgaagacccaggtgcagacccccagggcatgaaggagatcagcaacatccaggtgggcgacct
ggtgctgagcaacaccggctacaacgaggtgctgaacgtgttccccaagagcaagaagaagagctacaagatcaccctggaggacggca
aggagatcatctgcagcgaggagcacctgttccccacccagaccggcgagatgaacatcagcggcggcctgaaggagggcatgtgcctgt
acgtgaaggagatgatgctgaagaagatcctgaagatcgaggagctggacgagcgcgagctgatcgacatcgaggtgagcggcaaccac
ctgttctacgccaacgacatcctgacccacaacagcagcagcagcgacgtg

SopE
atgaccaagatcaccctgagcccccagaacttccgcatccagaagcaggagaccaccctgctgaaggagaagagcaccgagaagaaca
gcctggccaagagcatcctggccgtgaagaaccacttcatcgagctgcgcagcaagctgagcgagcgcttcatcagccacaagaacaccg
agagcagcgccacccacttccaccgcggcagcgccagcgagggccgcgccgtgctgaccaacaaggtggtgaaggacttcatgctgcag
accctgaacgacatcgacatccgcggcagcgcc

ER50
agcctggccctgagcctgaccgccgaccagatggtgagcgccctgctggacgccgagccccccatcctgtacagcgagtacgaccccacc
cgccccttcagcgaggccagcatgatgggcctgctgaccaacctggccgaccgcgagctggtgcacatgatcaactgggccaagcgcgtgc
ccggcttcgtggacctggccctgcacgaccaggtgcacctgctggagtgcgcctggatggagatcctgatgatcggcctggtgtggcgcagca
tggagcaccccggcaagctgctgttcgcccccaacctgctgctggaccgcaaccagggcaagtgcgtggagggcggcgtggagatattcga
catgctgctggccaccagcagccgcttccgcatgatgaacctgcagggcgaggagttcgtgtgcctgaagagcatcatcctgctgaacagcg
gcgtgtacaccttcctgagcagcaccctgaagagcctggaggagaaggaccacatccaccgcgtgctggacaagatcaccgacaccctgat
ccacctgatggccaaggccggcctgaccctgcagcagcagcaccagcgcctggcccagctgctgctgatcctgagccacatccgccacatg
agcagcaagcgcatggagcacctgtacagcatgaagtgcaagaacgtggtgcccctgagcgacctgctgctggagatgctggacgcccac
cgcctg

Supplementary Figure 2. DNA sequences of synthesized gene fragments.

Transcript	qPCR reaction	Primer sequence (5'-3')
UL27	SYBR green	GCTCAGAACCCCGTGCAAC
		GCAGAAGGTCTCCACGAACG
UL29	SYBR green	CATCTCATTGGCACGGTCTCG
		CAACTCGTACAGGCAGTCCTC
UL38	SYBR green	CCTACGACTCCGGTATCCTGT
		GTTCCAATACTCCAGCACGATAGC
UL117	SYBR green	CCCATGATCGACCTTACCA
		AATGTAGGTGGCGTTACCG
US2	SYBR green	CCTGCCCGATGGAATCACTAA
		CTTGCCGTTGTCAATGTAGCAC
US11	SYBR green	TCACGATTAAGTCGGCGCAGT
		AATGTCGGTGCAGCCAACCTT
US23	SYBR green	AGGTAATCCACGACGCCTTG
		ACGTTGTTTTCTTCGGGTTCCA
US24	SYBR green	TACAGCAGTTACACCGCATTTG
		GTCACGCCTAGCACATACCA
GAPDH	SYBR green	CTGTTGCTGTAGCCAAATTCGT
		ACCCACTCCTCCACCTTTGAC

Supplementary Table 1. Primer sequences used for RT-qPCR.
